# Caudal autotomy as anti-predatory behaviour in Palaeozoic reptiles

**DOI:** 10.1038/s41598-018-21526-3

**Published:** 2018-03-05

**Authors:** A. R. H. LeBlanc, M. J. MacDougall, Y. Haridy, D. Scott, R. R. Reisz

**Affiliations:** 1 Department of Biology, University of Toronto Mississauga, 3359 Mississauga Rd., Mississauga, Ontario L5L 1C6 Canada; 2 Department of Biological Sciences, University of Alberta, Alberta, T6G 2J5 Canada; 3 International Center of Future Science, Dinosaur Evolution Research Centre, Jilin University, Changchun, China

## Abstract

Many lizards can drop a portion of their tail in response to an attack by a predator, a behaviour known as caudal autotomy. The capacity for intravertebral autotomy among modern reptiles suggests that it evolved in the lepidosaur branch of reptilian evolution, because no such vertebral features are known in turtles or crocodilians. Here we present the first detailed evidence of the oldest known case of caudal autotomy, found only among members of the Early Permian captorhinids, a group of ancient reptiles that diversified extensively and gained a near global distribution before the end-Permian  mass extinction event of the Palaeozoic. Histological and SEM evidence show that these early reptiles were the first amniotes that could autotomize their tails, likely as an anti-predatory behaviour. As in modern iguanid lizards, smaller captorhinids were able to drop their tails as juveniles, presumably as a mechanism to evade a predator, whereas larger individuals may have gradually lost this ability. Caudal autotomy in captorhinid reptiles highlights the antiquity of this anti-predator behaviour in a small member of a terrestrial community composed predominantly of larger amphibian and synapsid predators.

## Introduction

Squamate caudal autotomy and subsequent tail regeneration have been studied extensively from ecological and developmental perspectives, because of the adaptive significance of this unique anti-predatory behaviour^1–5^. Extant lizards display two types of caudal autotomy: (1) intravertebral autotomy, where the caudal vertebrae are able to break apart along a pre-existing fracture plane that passes through the centrum and neural arch; and (2) intervertebral autotomy, where the split occurs between adjacent caudal vertebrae^2,4^. The former case is only seen in extant lepidosaurs and is associated with more extensive tail regeneration^1^. The latter case is found in several species of agamids and some species of snakes, and appears to have evolved multiple times from a non-autotomous ancestral condition^1,2^.

**Figure 1:**
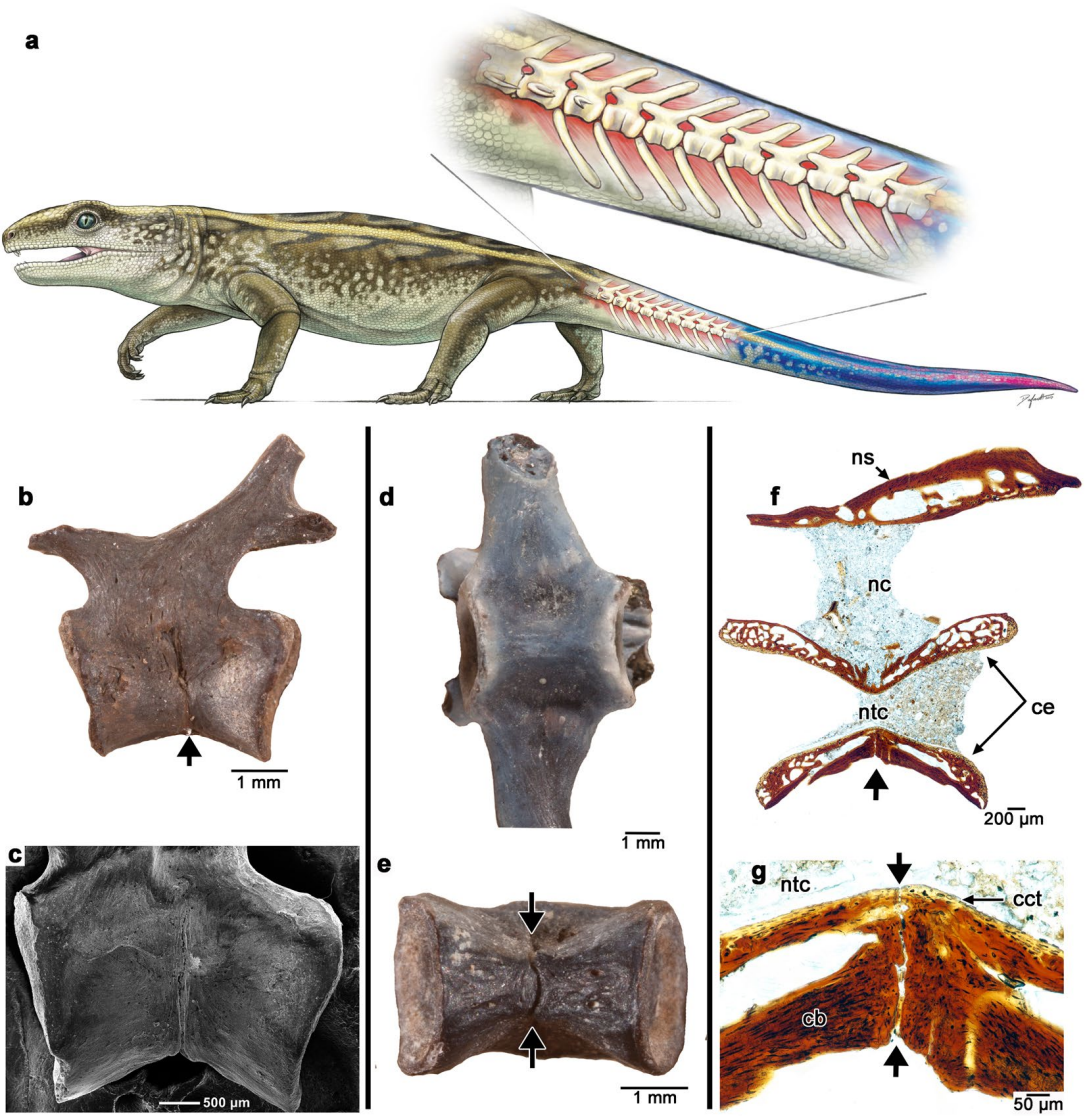
Fracture planes in captorhinid caudal vertebrae. (**a**) Artist’s reconstruction of the Permian reptile *Captorhinus* with an autotomous tail (inset showing anterior caudal vertebrae with fracture planes). (**b**) Image and (**c**) SEM image of an isolated anterior caudal vertebra (ROM 73769) with a fracture plane passing through the centrum (black arrow). (**d**) Ventral view of an anterior, rib-bearing caudal vertebra (ROM 77410) showing the absence of any fracture plane. (**e**) Ventral view of a caudal vertebra bearing a fracture plane (black arrows) (ROM 73771) (**f**) thin-section through the sagittal plane of a caudal vertebra (ROM 73773) with a fracture plane (black arrow) passing through the ventral portion of the centrum. (**g**) Close-up of fracture plane (black arrows) in (**f**) passing into the notochordal canal. Abbreviations: cb, cortical bone; cct, calcified cartilage; ce, centrum; nc, neural canal; ns, neural spine; ntc, notochordal canal. Reconstruction by Danielle Dufault. Anterior is to the left in all of the images.

Intravertebral autotomy probably represents the ancestral condition for Lepidosauria, because phylogenetically earlier families of lizards and the tuatara (*Sphenodon*) have fracture planes in their caudal vertebrae^1,3^. These fracture planes are areas of weakness along the vertebra resulting from incomplete fusion of adjacent sclerotomes during vertebral development^6^. In the fully ossified vertebra, the fracture plane is a transverse septum through the vertebra where no bone deposition occurs. Compared to the extensive studies of caudal autotomy in extant lepidosaurs, very little is known about caudal autotomy in extinct amniotes. Despite early reports of fracture planes in caudal vertebrae of aquatic mesosaurs^7^ and the early diapsid reptile *Araeoscelis*^8^, the only amniotes that show convincing evidence of caudal autotomy outside of Lepidosauria are members of the eureptilian clade Captorhinidae (Fig. 1)^9,10^.

Captorhinids were initially small carnivorous reptiles that originated in the Late Carboniferous and radiated extensively across the globe^11–13^, filling numerous ecological niches as relatively small-bodied carnivores, omnivores, and herbivores within terrestrial communities composed predominantly by predators^14–17^. The development, ontogenetic variation, and evolutionary significance of caudal autotomy in Palaeozoic reptiles have never been discussed beyond the preliminary reports of apparent fracture planes passing through several caudal vertebrae in three partial caudal series^9,10^ attributed to *Captorhinus* and *Labidosaurus*. Originally used to argue for eureptilian affinities of captorhinids^9^, advances in our understanding of reptilian phylogeny^18^ now clearly demonstrate that if captorhinids did indeed exhibit caudal autotomy, this was the earliest occurrence of this behaviour among Amniota, which later evolved independently in lepidosaurs.

The only records of fracture planes in the caudal vertebrae of captorhinids suggest that they do not pass completely through the centrum as they do in modern lepidosaurs^1,2,9^ and no detailed descriptions or comparisons to the autotomous vertebrae of extant lepidosaurs have ever been presented to test the autotomy hypothesis. Moreover, the potential for interspecific or ontogenetic variation in this feature across Captorhinidae has not been assessed. This is particularly important, because the number of putative autotomous vertebrae in captorhinids appears to vary drastically across different species^9,10^. These data are important for understanding how widespread this antipredatory behaviour was within early reptiles and if predation pressures varied, leading to ontogenetic changes in the capacity for autotomy for smaller and larger-bodied captorhinids, as occurs in some modern squamates^1^. To address these points, we examined a large sample of isolated captorhinid caudal vertebrae from the karst cave system at the Early Permian (Artinskian, 289–286 Ma^19^) Richards Spur locality, located at the Dolese Brothers Quarry in Oklahoma, U.S.A., which allowed for the first detailed anatomical and histological analysis of these structures and direct comparisons with the autotomous vertebrae in modern lizards. We compared putative fracture planes in captorhinids to those of juvenile and adult *Iguana iguana*, because this modern squamate exhibits ontogenetic reduction of caudal autotomy^2^. We then compared our sample to described captorhinid, synapsid, and parareptile caudal vertebrae from Richards Spur in order to better understand the extent of this feature within Early Permian amniotes.

## Results

### Captorhinid caudal vertebrae

For this study we limited our sample size to 70 isolated caudal vertebrae with presumed fracture planes collected from the Early Permian Richards Spur locality and three articulated caudal series attributed to the genus *Captorhinus* (Extended Data Table 1; Extended Data Fig. 1). These caudal vertebrae have transverse splits along the mid-ventral surfaces of the centra (Fig. 1a–c). These splits could not be taphonomic features, because they are consistently found in the same region of numerous caudal centra, are absent in more anterior rib-bearing caudal vertebrae (Fig. 1d), and possess straight edges and a rounded border along the ventral margin of the centrum (Fig. 1b,c,e). In all of the split-bearing vertebrae, these fracture planes extend from the ventral margins of the centra to the bases of the neural arches, but never extend on to the neural arches or spine, unlike the condition in most squamates^1,3,6^ (Fig. 1).

Longitudinal thin sections of autotomous caudal vertebrae in captorhinids reveal that the fracture plane extends through the ventral wall of the spool-shaped centrum and reaches the floor of the notochordal canal along the most dorsoventrally constricted part of the centrum (Fig. 1f,g). Similar to modern squamates^6,20^, each captorhinid caudal centrum is composed of three layers of hard tissue: calcified cartilage, endochondral/endosteal bone trabeculae, and compact cortical (periosteal) bone (Extended Data Fig. 1). Calcified cartilage forms the peripheries of the notochordal canal and gradually thickens towards the anterior and posterior ends of the centrum. The cell lacunae in the calcified cartilage are large, round, and contain very few canaliculi. Anteroposteriorly-oriented Sharpey’s fibers extend along the articular ends of the centrum (Extended Data Fig. 1g). The middle layer of bone of the centrum is composed of endochondral/endosteal trabeculae (sensu^20^). This trabecular bone layer forms the bulk of the centrum and is separated from the inner calcified cartilage and outer cortical bone by reversal lines, indicating that it is probably of endosteal origin closer to the outer bone cortex and endochondral bone where it replaces the calcified cartilage^20^.

**Figure 2:**
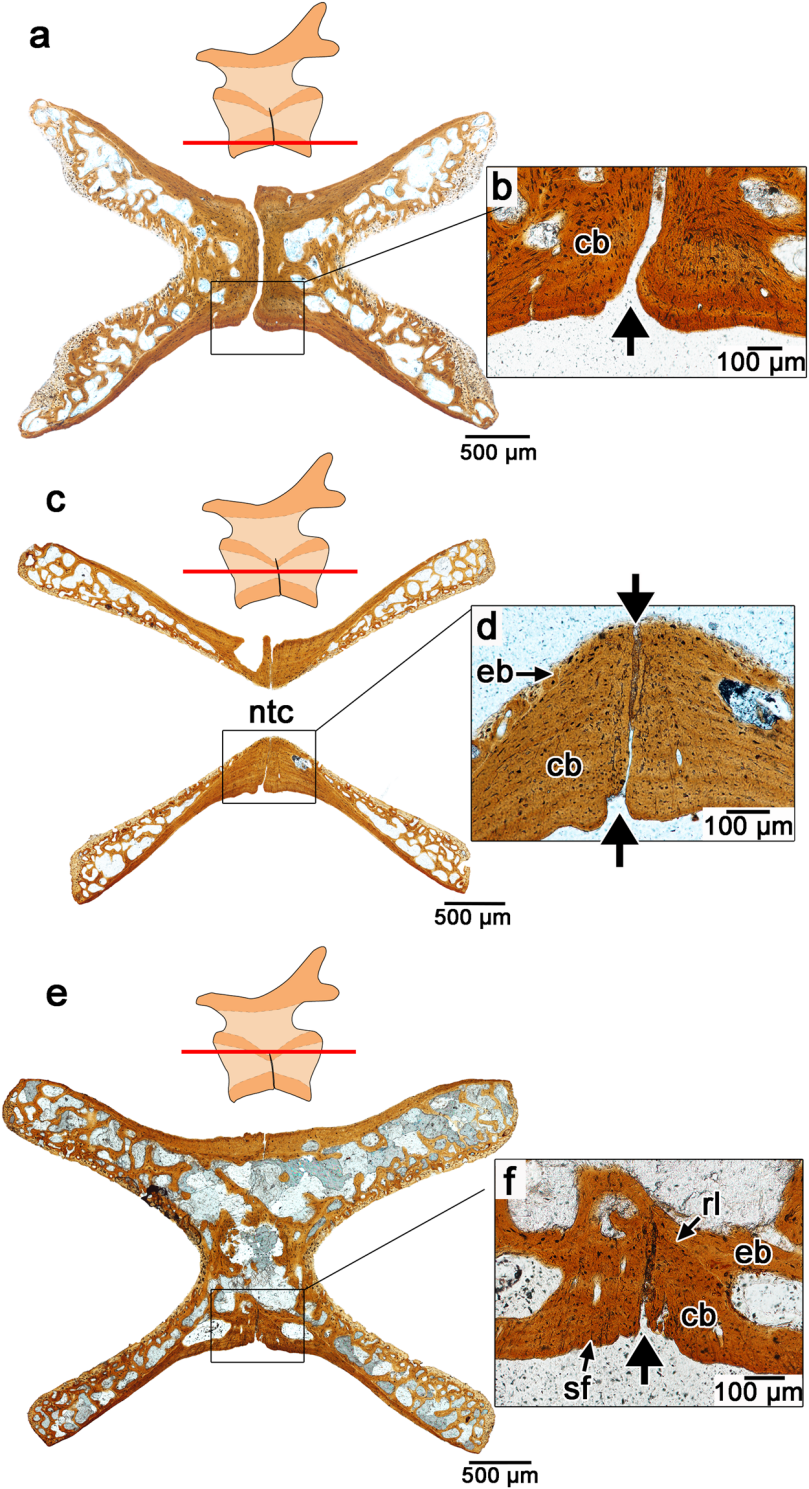
Serial transverse sections through the centra of fracture plane-bearing caudal vertebrae in captorhinids. (**a**) Transverse section taken near the base of the centrum (ROM 73771); (**b**) Closeup of the fracture plane running across the ventral surface of the centrum in (**a)**. (**c**) Transverse section taken at the level of the notochordal canal (ROM 73774). (**d**) Closeup of the fracture plane in **c** passing through the centrum into the notochordal canal. (**e**) Transverse section taken along the roof of the centrum (below the neural arch) (ROM 73774). (**f**) Closeup of fracture plane in (**g**) passing through the outer cortical bone, but not the more internal endosteal/endochondral bone. Abbreviations: cb, cortical bone; eb, endochondral bone; rl, reversal line; sf, Sharpey’s fibers. Anterior is to the left in all of the images.

The bone forming the outer shell of the centrum and surrounding the fracture plane is composed of compact cortical bone, which is thickest along the ventral border of the centrum. The cortical bone is organized into concentric layers of poorly vascularized, but highly cellular bone, reminiscent of parallel-fibered bone in the vertebrae of squamates^20^. The osteocyte lacunae are long, spindle-shaped, and contain numerous canaliculi (Fig. 1g). Sharpey’s fibers are abundant and extend dorsoventrally, indicating their roles as the anchoring fibers of the periosteum. The layers of cortical bone along the middle of the ventral portion of the centrum contour the fracture plane and show no evidence of bone resorption (Figs 1g and 2; Extended Data Fig. 1a,e), indicating that the fracture plane is bordered by primary bone. By comparison, longitudinal thin sections through an anterior rib-bearing caudal vertebra show that the cortical bone forming the ventral edge of the centrum is uniform, showing no evidence of a lack of ossification between the anterior and posterior halves of the centrum (Extended Data Fig. 1c,d,f).

These comparisons indicate that the split forms during ossification of the centrum and not as a result of bone resorption along the midline of the centrum. Autotomy septa are similarly formed during the ossification of the caudal vertebrae and not by bone resorption in modern squamates^6^. A thin wall of cortical bone completes the dorsal wall of the notochordal canal. Consequently, the only portion that connects the anterior and posterior halves of these caudal vertebrae is the dorsal mid-centrum surface and the fused neural arches, which fuse later in ontogeny. The dorsal surface of the centrum bordering the notochordal canal is extremely thin, being only 50–100 μm thick in vertebrae that are over 3 mm long.

Serial transverse sections through fracture plane-bearing caudal vertebrae confirm that these features are restricted to the ventral half of each centrum. The gap between anterior and posterior halves of the centrum is considerable at the ventral base of the vertebra (Fig. 2a,b). Transverse sections through the middle portion of the centrum show that the fracture plane still passes through the entire width of the lateral walls of the centrum and into the notochordal canal (Fig. 2c,d). Transverse sections taken near the base of the neural arch still show evidence of fracture planes along the lateral margins of the centra, but the endochondral/endosteal bone forming the internal core of the vertebra interrupts these planes (Fig. 2e,f).

**Figure 3:**
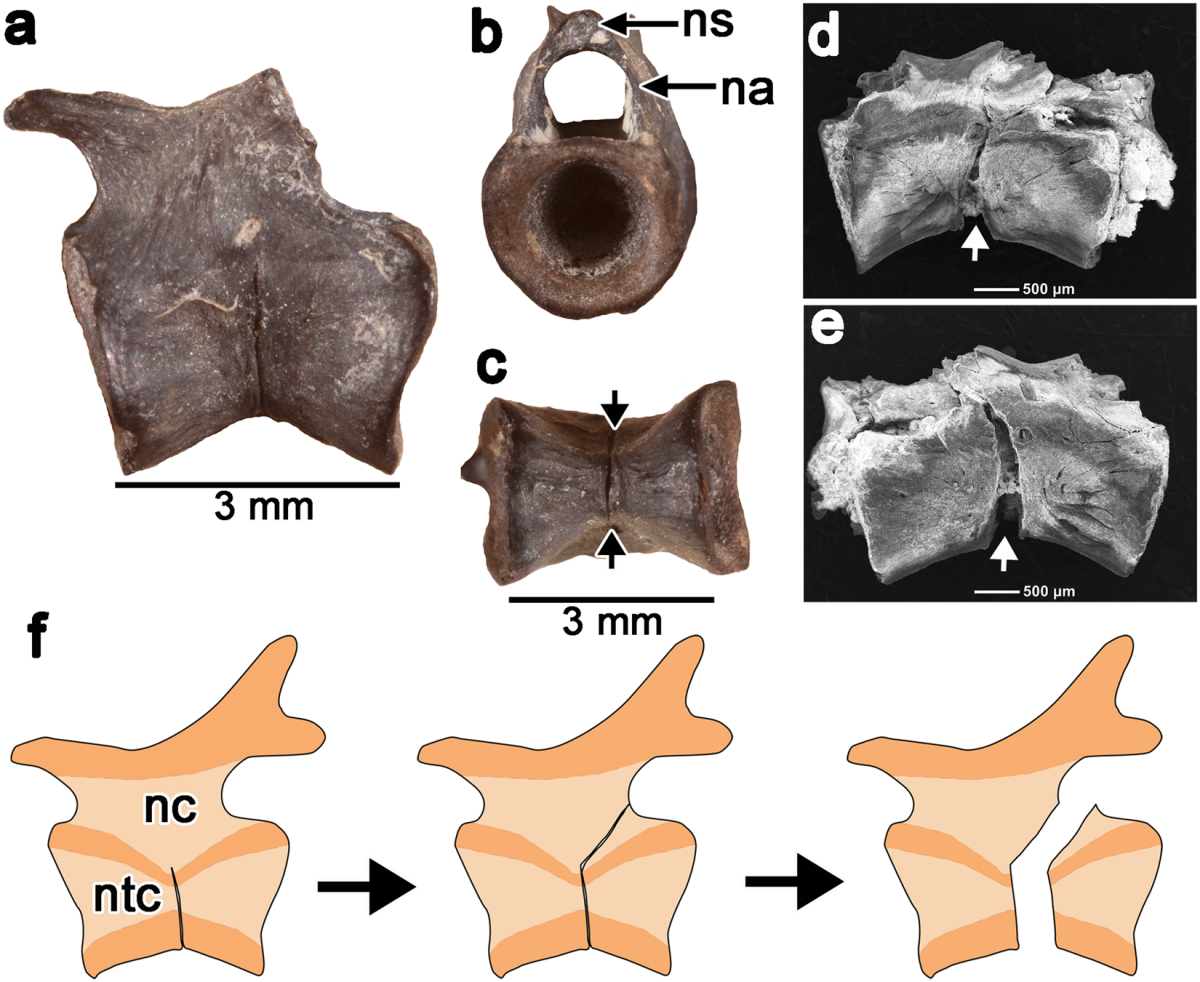
Hypothesized path of autotomy break in the caudal centra of captorhinids. (**a**–**c**) Lateral, anterior, and ventral views of a fracture plane-bearing caudal centrum, showing the extend of the fracture plane, which does not pass through the neural arch or spine (ROM 73774). (**d**,**e**) SEM images of a post mortem break in a captorhinid caudal vertebra (ROM 77409) along its fracture plane. The break follows the plane of weakness formed by the autotomy plane (white arrow) and is found on the left (**d**) and right (**e**) sides, suggesting that this was the plane of weakness in life. (**f**) Reconstruction of caudal autotomy in a caudal vertebra. During autotomy, the fracture follows the path of least resistance through the dorsal half of the caudal centrum and the posterior base of the neural arch, bypassing the neural spine. Abbreviation: na, neural arch; nc, neural canal; ns, neural spine; ntc, notochordal canal. Anterior is to the left in all of the images.

Despite being restricted to the ventral half of each caudal centrum, the fracture planes probably still allowed for caudal autotomy. One vertebra exhibits apparent post-mortem breakage along the fracture plane, providing a glimpse of the dynamics of intravertebral autotomy in captorhinids. The vertebra clearly exhibits a fracture plane, based on the rounded edges on either side of the break along the ventral surface of the centrum (Fig. 3). The subsequent break must have occurred some time after death and disarticulation of the remains of this individual. Nevertheless, the crack follows the fracture plane dorsally and extends slightly posteriorly towards the posterior end of the neural arch. This break is present on both sides of the centrum, terminates posterior to the neural spine, and extends posteriorly past the neural arch, thus allowing caudal vertebrae with fused neural arches to still be able to break apart (Fig. 3).

**Figure 4:**
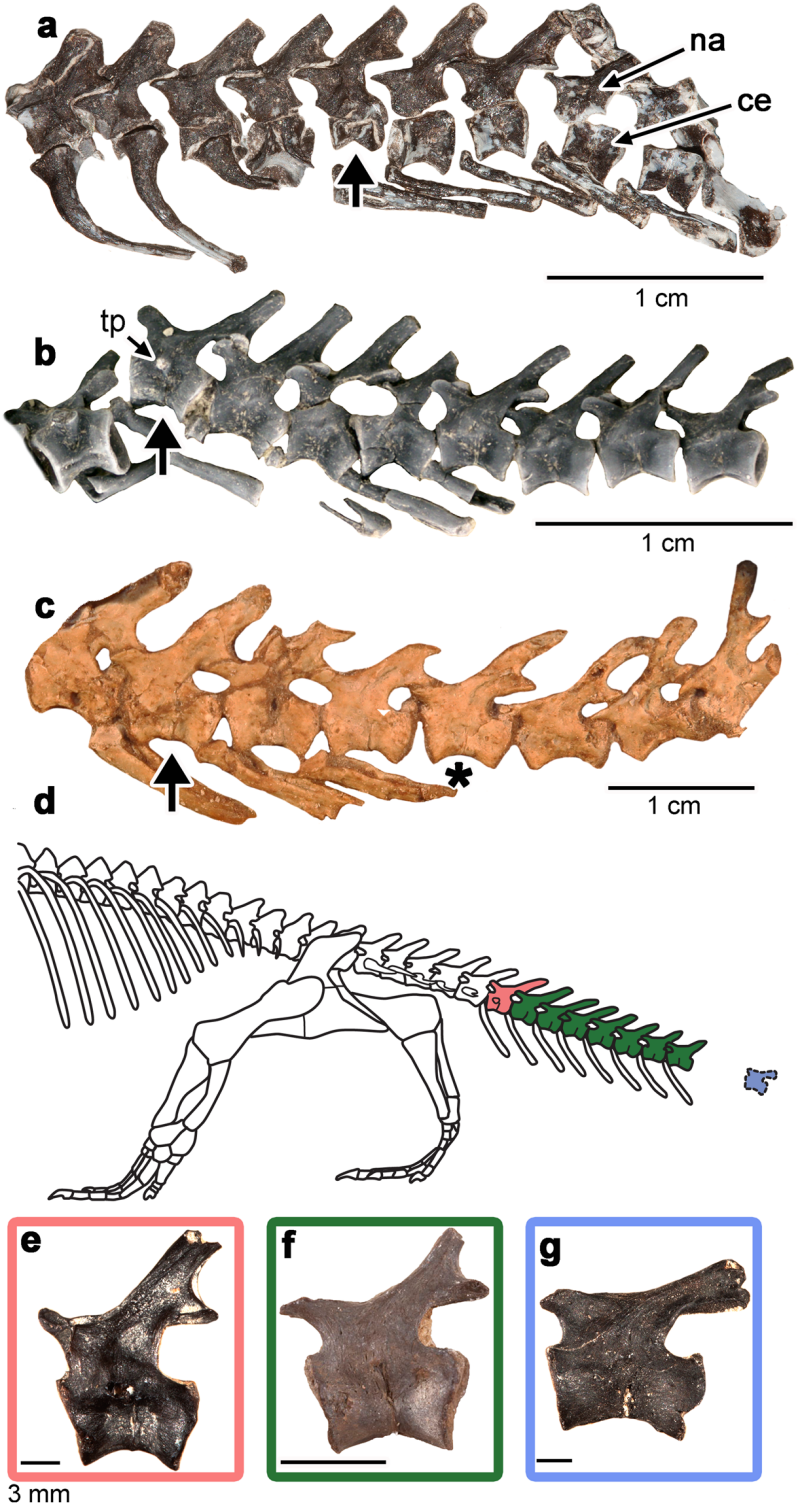
Variation in autotomous caudal regions in Early Permian captorhinids. (**a**) Partial caudal series of a juvenile captorhinid (OMNH 03304) with at least five autotomous vertebrae, beginning with the last transverse process-bearing caudal (arrow). Note the presence of unfused neural arches. (**b**) Autotomous region of an articulated caudal series (OMNH 1020, the image has been cropped to include only the caudal series) with at least eight autotomous vertebrae, beginning with the last transverse process-bearing caudal (arrow). (**c**) Partial caudal series of *Captorhinus laticeps* (OUSM 15024, the image has been cropped to include only the caudal series) with an autotomous region beginning at the eighth caudal (arrow) and ending at the eleventh (asterisk). (**d**) Reconstruction of the known caudal region of *Captorhinus* (modified from)^22^, showing extent of autotomous region (coloured) based on (**a**–**f**). (**e**–**g**) Isolated anterior (transverse process-bearing), middle (lacking transverse processes, neural spines steeply inclined), and posterior (low-angled neural spines) caudal vertebrae of captorhinids from the Richards Spur locality. Abbreviation: tp, transverse process. Anterior is to the left in all of the images.

The precise number of caudal vertebrae in *Captorhinus*, the most abundant captorhinid at the Richards Spur locality, is unknown^10,21^. Furthermore, the caudal vertebrae of captorhinids are fairly uniform in shape posterior to the last rib-bearing caudal^21^, making it difficult to determine if some of the extremely small centra belonged to anterior caudals of young individuals, or to more posterior regions of the tail of larger species or individuals. However, some vertebrae can be confidently assigned to anterior, middle, and posterior regions of the tail based on preserved articulated caudal series of captorhinids (Fig. 4a–c). Some transverse process-bearing anterior caudal vertebrae have fracture planes and correspond to the sixth to eighth caudal based on articulated caudal series of *Captorhinus aguti* and *C. laticeps* (Fig. 4a–d)^10,21,22^. The neural spines of the anterior caudals are tall and narrow and extend posterodorsally at relatively high angles. Their respective centra are proportionally short and bevelled as a result of the articulation with the haemal arches. More posterior caudals have longer, spool-shaped centra, lack transverse processes, and the neural spines extend at much shallower angles posterodorsally (Fig. 4e–g). These mid-caudal vertebrae frequently possess fracture planes, indicating that a large portion of the tail was autotomous. Unfortunately, the precise number of autotomous vertebrae cannot be determined, but our examination of three partial caudal series of *Captorhinus* reveals at least eight autotomous vertebrae, beginning around the sixth to eighth caudal (Figs 1a and 4a–c). Moreover, the elongate spool shapes and low-angled neural spines of several of the isolated caudals we examined indicate that many of the isolated autotomous vertebrae are from even farther posteriorly along the tail (Fig. 4g). Eight autotomous caudal vertebrae is therefore a very conservative estimate for the Richards Spur captorhinids. Interestingly, a caudal series of a large specimen of *Captorhinus laticeps* from the McAnn quarry of Oklahoma (Fig. 4c) shows a much shorter autotomous section of the tail, which is limited to only four anterior caudals.

### Iguanid caudal vertebrae

**Figure 5:**
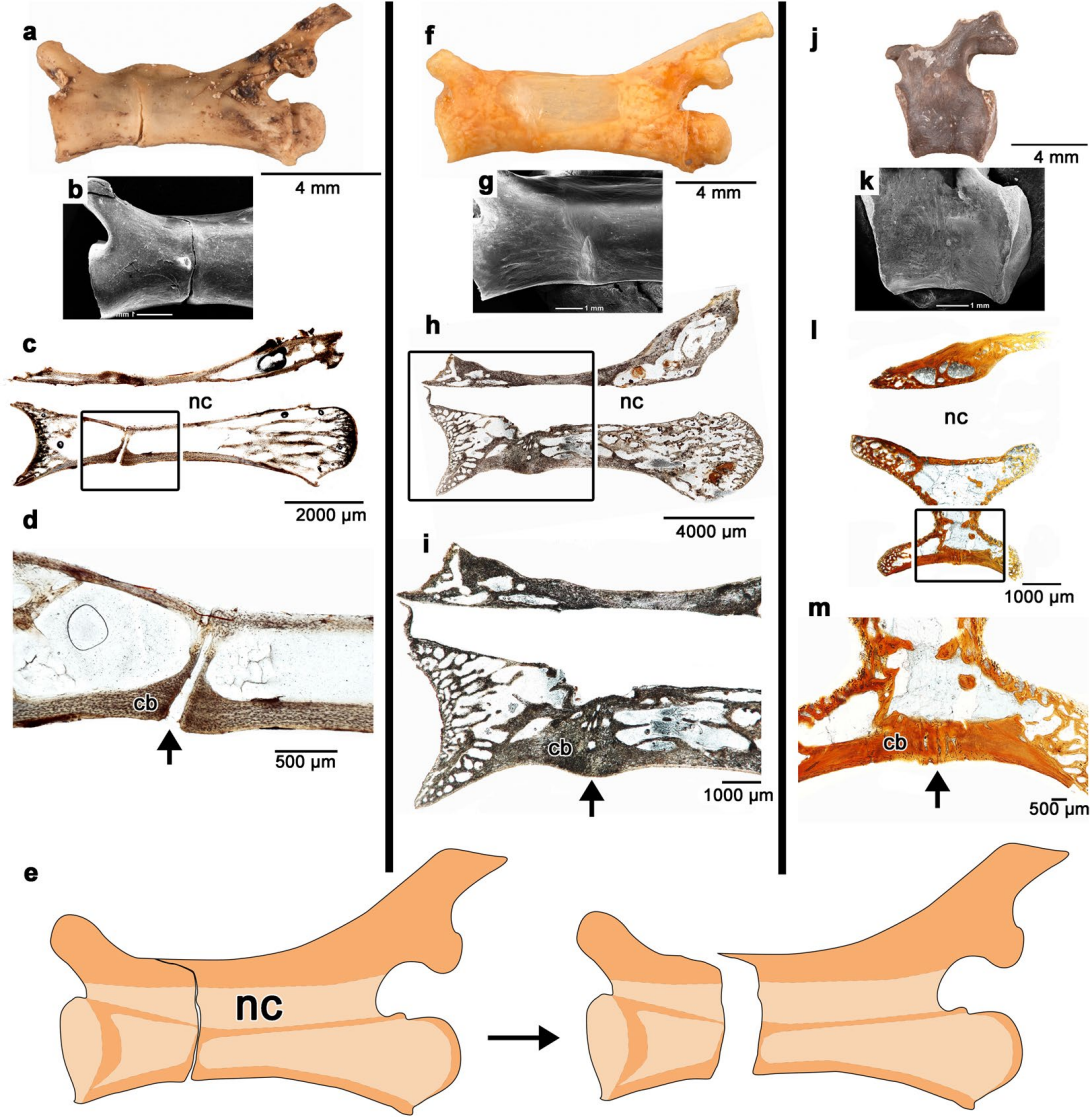
Comparisons of fracture planes through ontogeny in squamates and captorhinids. Caudal vertebrae in juvenile *Iguana* (ROM R9175) (**a**–**d**) have fracture planes that extend through the entire centrum in thin section. During autotomy, these fracture planes facilitate a break in the anterior third of the vertebrae, through the neural arch and spine, to allow the vertebra to split in two (**e**). Older, larger *Iguana* (ROM 77408) (**f**–**i**) have closed fracture planes and show less frequency of autotomy^1^. Large anterior caudal vertebrae of captorhinids (ROM 73770) (**j**–**m**) similarly possess closed fracture planes in thin section. Abbreviations: cb, cortical bone; nc, neural canal. Anterior is to the left in all of the images.

The anatomy and development of autotomous caudal vertebrae in squamates have been described extensively^1,6,23^. We compared our sample of fracture plane-bearing caudal vertebrae in captorhinids to the autotomous vertebrae of juvenile and adult specimens of extant *Iguana iguana* in the osteological collections at the Royal Ontario Museum in Toronto, Canada (ROM R9175, 77408). Unlike the condition in captorhinids, the fracture planes in the juvenile *Iguana* (ROM R9175) pass through the entire centrum and nearly the entire neural arch (Fig. 5a–d). In thin section, iguanid vertebral centra are similarly formed by calcified cartilage at the anterior and posterior ends, internal bony trabeculae of endochondral and endosteal origin, and an outer shell of compact cortical bone^6,20^ (Fig. 5c,d). The fracture plane is bordered by compact cortical bone, similar to the fracture planes in captorhinids. In juvenile *Iguana*, the fracture plane is largest at the ventral margin of the centrum and passes nearly to the neural canal (Fig. 1d). Longitudinal sections along the midline of the caudal vertebrae show a thin layer of cortical bone at the dorsal extremity of the fracture plane that appears to hold the two halves of the caudal centrum together (Fig. 1d). During autotomy, this split allows the vertebra to break apart, leaving a short anterior portion of the centrum and neural arch (Fig. 5e). In the larger individual, the autotomy septum is closed by a mass of compact cortical bone and is barely visible along the outer surface of the centrum (Fig. 5f–i).

Some captorhinid caudal vertebrae show external evidence of a fracture plane along the surface of the centrum, but the plane does not pass through the ventral surface of the bone in thin section (Fig. 5j–m). The mid-ventral region of the centrum is not smooth, as in the anterior caudal vertebrae that lack fracture planes (Extended Data Fig. 1f), but instead possesses a mass of poorly organized, vascularized cortical bone in this region, suggesting that, similar to the larger caudal vertebra of *Iguana*, may represent a closed fracture plane.

### Parareptile and synapsid caudal vertebrae from Richards Spur

In order to determine if fracture planes are exclusive to captorhinids at Richards Spur, we examined described and recently prepared caudal vertebrae of parareptiles and synapsids from the locality. Currently, very few parareptilian caudal vertebrae are known from Richards Spur, however, we found and prepared the caudal series of an unidentified juvenile parareptile (Extended Data Fig. 2). This caudal series of seven vertebrae is interpreted as parareptilian due to the presence of paired ventral ridges on the centra, a characteristic that is observed in other parareptiles at the locality^24^. The vertebrae in this series lack fracture planes on their centra (Extended Data Fig. 2), suggesting that they would not be able to undergo autotomy. Furthermore, there are no synapsid caudal series known from Richards Spur, however, a single anterior caudal of the caseid *Oromycter dolesorum* is known^25^. This isolated vertebra possesses a centrum that lacks a fracture plane, clearly indicating that it would not have exhibited any intervertebral autotomy, this likely would have extended to the rest of the anterior caudals as well.

## Discussion

Our anatomical and histological examination of captorhinid caudal vertebrae from the Richards Spur locality supports previous hypotheses^9,10^ that *Captorhinus* and other early Permian captorhinids had the ability to drop a significant portion of their tail as a predator escape strategy. Similar to modern lepidosaurs, fracture plane-bearing caudal vertebrae begin at the sixth to eighth position, posterior to the insertions for the *m. caudofemoralis longus*, a vital muscle for hindlimb retraction^4,26^. These fracture planes are also absent along the cervical, dorsal, and sacral series^21,22^ and therefore must have exclusively functioned as autotomy septa. Despite being restricted to the ventral half of the centrum in captorhinids, the fracture planes clearly served a role in caudal autotomy, particularly in juveniles, where the neural arches are not fused to the centra (Fig. 4a). The spool-shaped vertebrae of captorhinids are tightly constricted at the mid-point of the centrum (Fig. 1e), and coupled with the presence of a fracture plane, probably allowed for autotomy even when the neural arches were fused to the centra. Unlike iguanids (but similar to gekkotans and *Sphenodon*^23^) captorhinid caudal vertebrae consisted of an open notochordal canal and therefore only thin layers of bone held the anterior and posterior halves of the vertebrae together along the dorsal wall of the centrum (Fig. 1f).

Taphonomic breaks to one captorhinid caudal vertebra (Fig. 3) show that the fracture planes may have served to propagate a fracture along the base of the neural arch when a predator grasped the tail. The fracture would progress posterior to any transverse processes and the bulk of the neural spine (Fig. 3f). This would have allowed a caudal centrum to break into two unequal halves during autotomy, even when the neural arches were fused to the centra. This form of autotomy would not have resulted in a precise break through a single vertebra, but would nevertheless be effective in dropping the tail during an attack by a predator. Similarly, Seligmann *et al*.^3^ showed that the sites of caudal autotomy do not always precisely follow the fracture planes in the caudal vertebrae of *Sphenodon*, suggesting that these types of fracture planes serve as the pathways of least resistance when forces are applied to drop the tail, a function we extend to the fracture planes in captorhinids.

To date, there is no evidence to suggest that captorhinids were able to regenerate a lost portion of their tail in any comparable way to modern lepidosaurian species. It is interesting to note, however, that among lepidosaurs, tail regeneration is most strongly associated with intravertebral caudal autotomy^1,2,23^ and the regenerated cartilaginous cone that replaces the lost caudal vertebrae is sometimes preserved in fossils^27^. The preservational environment at Richards Spur did facilitate the preservation of articulated material^19^, however the potential to preserve a regenerated cartilage cone is currently unknown. If captorhinids regenerated the tail following autotomy in a similar fashion to modern lepidosaurs, then some anterior fragments of caudal vertebrae may also show signs of extensive bone resorption, which would indicate the initial stages of wound healing and formation of the cartilage cone of the regenerated tail^3,23^. However, we did not identify any such vertebrae in our sample.

The differences in size and degree of ossification (fused versus unfused neural arches) of the sample of autotomous vertebrae from Richards Spur suggest that the ability to autotomize the base of the tail is likely common to multiple species of captorhinid at the locality, and that there was ontogenetic variability in the retention of caudal autotomy. Concerning the former possibility, there are six captorhinomorph species currently known from Richards Spur, many of which have roughly equal body size ranges^14,28–31^. Unfortunately, the lack of complete caudal skeletons for these species makes it difficult to identify which captorhinid species exhibited caudal autotomy. However, all known captorhinid articulated anterior caudal series from this locality exhibit autotomy or closed fracture planes. We also found numerous isolated caudal vertebrae with fracture planes from various pockets within the vast cave system at Richards Spur, likely an indication that multiple species of captorhinids at this locality exhibited caudal autotomy.

The majority of the fossiliferous pockets at the Dolese Brothers quarry contain mainly the remains of the smaller captorhinid species, including *Captorhinus aguti*, but a distinct, deeper pocket that mainly yielded remains of the larger species *C. magnus*^28^ also yielded numerous caudal vertebrae with fracture planes. Unfortunately, the nature of the cave infillings and the ongoing quarrying operations make it difficult to determine the stratigraphic context for any of the material collected from the site^19,28^. However, fracture planes were observed in captorhinid caudal vertebrae from partially articulated caudal series, white and black (hydrocarbon-stained) bone, as well as isolated and weathered caudal vertebrae, thus spanning most of the taphonomic modes, and presumably the different fossiliferous pockets at the locality^19^. Fracture planes are also found in articulated caudal series of *Captorhinus laticeps* from the McCann Quarry in Oklahoma (Fig. 4c), as well as the more distantly related captorhinid *Labidosaurus* from Texas^7,9,10^, indicating that caudal autotomy was prevalent across Captorhinidae.

One of the examined caudal series clearly belongs to a juvenile individual, as indicated by the lack of fusion of the neural arches to the centra^32^ and possesses a minimum of five autotomous vertebrae (Fig. 4a). Another caudal series from a slightly more osteologically mature individual (with fused neurocentral sutures) possesses a minimum of eight autotomous caudal vertebrae (Fig. 4b). By comparison, the autotomous caudal series in a relatively large specimen of *Captorhinus laticeps* (Fig. 4c) consists of only four vertebrae, with the subsequent three vertebrae clearly lacking fracture planes. This suggests that there may be ontogenetic or taxic variability in the number of caudals with fracture planes. We also found several isolated vertebrae from Richards Spur that possessed closed fracture planes. A histological thin section of one of these vertebrae reveals a fracture plane along the ventral border of the centrum that is closed at its ventral margin by highly vascularized cortical bone (Fig. 5). Juvenile iguanas have open fracture planes extending through the centrum that are completely enclosed by cortical bone in adults (Fig. 5). These comparisons also indicate that at least some captorhinids exhibit ontogenetic variation in the ability to autotomize their tails, gradually shortening the autotomous region to a narrow portion of the anterior part of their tails through ontogeny. Interestingly, ontogenetic reduction in caudal autotomy in modern squamates follows this pattern, where fracture planes are closed by bone growth first in more posterior caudal vertebrae, and proceeds gradually to more anterior vertebrae^1,2^. This pattern of closure of the fracture planes may explain why the caudal series in *Captorhinus laticeps* is restricted to only four anterior caudal vertebrae.

Finally, our model, with the fracture plane running through the centrum, and then passing backwards posterior to the neural spine appears to be the most parsimonious explanation for how the vertebrae were able to break apart. However, unlike in lizards, that would mean that despite breaking the centrum in half, there would still be a connection between the zygapophyses of the neural arches. Even in juvenile captorhinids where the neural arch is not fused to the centrum, our model would require a further break between the zygapophyses of adjacent vertebrae. Thus, captorhinids appear to have a unique type of caudal autotomy, a mix between intra- and intervertebral events. The bulk of the break occurs through the body of the caudal centrum (which is consistent with intravertebral autotomy in lepidosaurs), with the final break occurring somewhere between the zygapophyses, possibly similar to intervertebral autotomy in some extant squamates^2,4^.

## Conclusions

Caudal autotomy can increase the chances of survival by facilitating escape, distracting predators, and minimizing blood loss following injury^33^. This anti-predator behaviour is prevalent among extant, small-bodied lepidosaurs; more than half of all families of modern squamates use caudal autotomy as an escape strategy^4,5^. Despite its prevalence within Squamata^1^, extensive variation exists in the positions of fracture planes along or between each vertebra, as well as in the number and positions of autotomous vertebrae^1,34^. In these respects, captorhinids were most similar to some iguanids and the extant lepidosaur *Sphenodon*^3,5^. As in these taxa, captorhinid caudal vertebrae possess fracture planes along the middle of the centra, beginning with the sixth to eighth caudal vertebra, at the position of the last transverse process-bearing vertebra, and continuing for at least seven more posteriorly. However, there is convincing evidence that the autotomous region probably includes several more vertebrae (Fig. 4g). This means that many captorhinids were able to drop significant portions of their tails either to distract would-be predators or to escape a predator’s grasp. The frequency of tail loss in modern squamates correlates strongly negatively with the distance of the autotomous region from the cloaca^35^, suggesting that the beginning of the autotomous vertebral region near the base of the tail may reflect a high frequency of this behaviour as a predator escape strategy in populations of Early Permian captorhinids.

Many Early Permian captorhinids were small-bodied tetrapods (less than 10 kg^16^) that were abundant members of carnivore-dominated assemblages^15,36,37^. The prevalence of caudal autotomy in captorhinids from the Early Permian Richards Spur locality implicates strong selection pressures favouring such anti-predatory behaviours, particularly for small-bodied captorhinids. Indeed, the Richards Spur tetrapod assemblage is similar to most Early Permian fossil assemblages in that it contains numerous carnivorous taxa and very few herbivores^16,36,37^. The Richards Spur captorhinids were likely predated upon by larger carnivores, including the anamniotes *Acheloma*^38^ and *Cacops*^39^, varanopid and sphenacodontid synapsids^40,41^, even possibly by larger captorhinids. In such carnivore-dominated assemblages, small-bodied taxa may have experienced significantly higher predation pressures and thus would have favoured the evolution of anti-predatory behaviours, including caudal autotomy. Other amniotes at Richards Spur, including small caseids, do not exhibit any evidence of caudal autotomy^25^. Notably, an articulated caudal series of a young parareptile from Richards Spur also does not show any indication of fracture planes (Extended Data Fig. 2), suggesting that these similarly-sized reptiles did not autotomize their tails. Thus, this behaviour was likely restricted to the captorhinids of the locality. All the available evidence^42,43^, including a thorough examination of all known articulated skeletons with well preserved caudal vertebrae (R. R. R., pers. obs.), leads us to conclude that captorhinids are thus far the only Palaeozoic amniotes known to have evolved this anti-predator behaviour. Caudal autotomy may thus have played an important role in the early diversification of captorhinids, as they were the first amniote group to achieve a near global distribution and diversify into new ecological niches in the Late Palaeozoic^12,13,15,17,44^.

## Materials and Methods

We examined 70 isolated or pairs of captorhinid caudal vertebrae from the Royal Ontario Museum (Toronto, Canada) collections (ROM 75623–75689, 73769–73774, 77409, 77409) and three partial articulated caudal series from the Sam Noble Oklahoma Museum of Natural History and the University of Oklahoma Stoval Museum (Norman, Oklahoma) that showed evidence of fracture planes (OMNH 1020, 03304, OUSM 15024). We also used an anterior rib-bearing caudal vertebra for comparative thin sections (ROM 77410). Several specimens were examined using a Jeol Scanning Electron Microscope (SEM). Seven caudal vertebrae of *Captorhinus* from the Early Permian Richards Spur locality were thin-sectioned for histological analyses (Extended Data Table 2) following the ROM procedure for sectioning fossil material. Specimens were embedded in Castolite AC polyester resin and placed under vacuum. Thin sections were processed using a low-speed Buehler Isomet wafer blade saw and fixed to plexiglass slides using cyanoacrylate. Mounted blocks were cut again on the Isomet saw and ground down using a Hillquist grinding machine. Samples were then ground by hand using 600- and 1000-grit silicon carbide powder on glass plates to the desired thickness. Images were taken on a Nikon AZ-100 microscope, NIS Elements BR software, and a Nikon Ds-Fi2 camera registered to R.R. Reisz. Most of the vertebrae correspond to anterior caudals, based on the anteroposteriorly short spool-shaped centra and tall neural spines. Two autotomous caudal vertebrae of the squamate *Iguana* (ROM R9175, 77408) were sectioned histologically for comparative purposes. Both were taken from skeletonized and disarticulated specimens, but from roughly equivalent positions along the tail (the juvenile vertebra, ROM R9175 was taken from slightly more anteriorly, due to the presence of a small transverse process). These sections were made following the same procedures as the fossil material and no stains were used. Furthermore, a series of caudal vertebrae belonging to an unidentified juvenile parareptile from the Richards Spur locality (ROM 77622) was examined (Extended Data Fig. 2).

## Electronic supplementary material

Supplementary Data and Figures
