# Supplementary material for: Caudal autotomy as anti-predatory behaviour in Palaeozoic reptiles
Source: Sci Rep. 2018 Mar 5;8:3328. doi: 10.1038/s41598-018-21526-3 (PMC5838224; doi:10.1038/s41598-018-21526-3)
Supplement: Supplementary file 1 — Supplementary Data and Figures [file 41598_2018_21526_MOESM1_ESM.pdf]

Caudal autotomy as anti-predatory behaviour in Palaeozoic reptiles

A. R. H. LeBlanc, M. J. MacDougall, Y. Haridy, D. Scott, R. R. Reisz

Supplementary Information

Extended Data Table 1. Captorhinid caudal vertebrae examined in this study

Number	Description	Size[1]
ROM 75623	Anterior caudal with fracture plane	4 mm
ROM 75624	Anterior caudal with fracture plane	3 mm
ROM 75625	Anterior caudal with fracture plane	3 mm
ROM 75626	Anterior caudal with fracture plane	3 mm
ROM 75627	Anterior caudal with fracture plane	5 mm
ROM 75628	Anterior caudal with fracture plane	4 mm
ROM 75629	Anterior caudal with fracture plane	5 mm
ROM 75630	Anterior caudal with fracture plane	5 mm
ROM 75631	Anterior caudal with fracture plane	4 mm
ROM 75632	Anterior caudal with fracture plane	6 mm
ROM 75633	Anterior caudal with fracture plane	4 mm
ROM 75634	Anterior caudal with fracture plane	4 mm
ROM 75635	Anterior caudal with fracture plane	6 mm
ROM 75636	Anterior caudal with fracture plane	4 mm
ROM 75637	Anterior caudal with fracture plane	5 mm
ROM 75638	Anterior caudal with fracture plane	4 mm
ROM 75639	Anterior caudal with fracture plane	4 mm
ROM 75640	Anterior caudal with fracture plane	5 mm
ROM 75641	Anterior caudal with fracture plane, transverse processes present	7 mm
ROM 75642	Anterior caudal with fracture plane	5 mm
ROM 75643	Anterior caudal with fracture plane	6 mm
ROM 75644	Anterior caudal with fracture plane	3 mm
ROM 75645	Anterior caudal with fracture plane	4 mm
ROM 75646	Anterior caudal with fracture plane	4 mm
ROM 75647	Anterior caudal with fracture plane	4 mm
ROM 75648	Anterior caudal with fracture plane	4 mm
ROM 75649	Anterior caudal with fracture plane	4 mm
ROM 75650	Anterior caudal with fracture plane	5 mm
ROM 75651	Anterior caudal with fracture plane	4 mm
ROM 75652	Anterior caudal with fracture plane	4 mm
ROM 75653	Anterior caudal with fracture plane	5 mm
ROM 75654	Anterior caudal with fracture plane	4 mm
ROM 75655	Anterior caudal with fracture plane	3 mm
ROM 75656	Anterior caudal with fracture plane	3 mm
ROM 75657	Anterior caudal with fracture plane	4 mm
ROM 75658	Anterior caudal with fracture plane	3 mm
ROM 75659	Anterior caudal with fracture plane	4 mm
ROM 75660	Anterior caudal with fracture plane	4 mm

ROM 75661	Anterior caudal with fracture plane	3 mm
ROM 75662	Anterior caudal with fracture plane	3 mm
ROM 75663	Anterior caudal with fracture plane	2 mm
ROM 75664	Anterior caudal with fracture plane	3 mm
ROM 75665	Anterior caudal with fracture plane	4 mm
ROM 75666	Anterior caudal with fracture plane	3 mm
ROM 75667	Anterior caudal with fracture plane	4 mm
ROM 75668	Anterior caudal with fracture plane	4 mm
ROM 75669	Anterior caudal with fracture plane	3 mm
ROM 75670	Anterior caudal with fracture plane	4 mm
ROM 75671	Half an anterior caudal, split at the fracture plane	2 mm
ROM 75672	Anterior caudal with fracture plane	3 mm
ROM 75673	Anterior caudal with fracture plane	5 mm
ROM 75674	Series of two anterior caudals with fracture planes	7mm
ROM 75675	Anterior caudal with fracture plane	3 mm
ROM 75676	Anterior caudal with fracture plane	3 mm
ROM 75677	Anterior caudal with fracture plane	6 mm
ROM 75678	Anterior caudal with fracture plane	8 mm
ROM 75679	Anterior caudal with fracture plane	6 mm
ROM 75680	Anterior caudal with fracture plane	4 mm
ROM 75681	Series of two anterior caudals with fracture planes	10 mm
ROM 75682	Anterior caudal with fracture plane, transverse processes present	6 mm
ROM 75683	Anterior caudal with fracture plane	7 mm
ROM 75684	Anterior caudal with fracture plane	3 mm
ROM 75685	Anterior caudal with fracture plane, transverse process on left side	6 mm
ROM 75686	Anterior caudal with fracture plane	4 mm
ROM 75687	Anterior caudal with fracture plane	4 mm
ROM 75688	Anterior caudal with fracture plane	6 mm
ROM 75689	Series of two pathological caudals with fracture planes	6 mm
ROM 77409	Anterior caudal with break along fracture plane	4 mm
OMNH 1020	Partial caudal series of a juvenile captorhinid	3 cm
OMNH 03304	Partial dorsal and caudal series of a captorhinid	3 cm
OUSM 15024	Partial caudals series of *Captorhinus laticeps*	4 cm

[1]Anteroposterior length of the vertebra, or vertebrae if they are a series.

Extended Data Table 2. Vertebrae that were histologically sectioned in this study.

ROM 73769	Anterior caudal with a fracture plane
ROM 73770	Anterior caudal with a fracture plane
ROM 73771	Anterior caudal with a fracture plane
ROM 73772	Anterior caudal with a fracture plane
ROM 73773	Anterior caudal with a fracture plane
ROM 73774	Anterior caudal with a fracture plane
ROM 77410	Anterior, rib bearing caudal without fracture plane

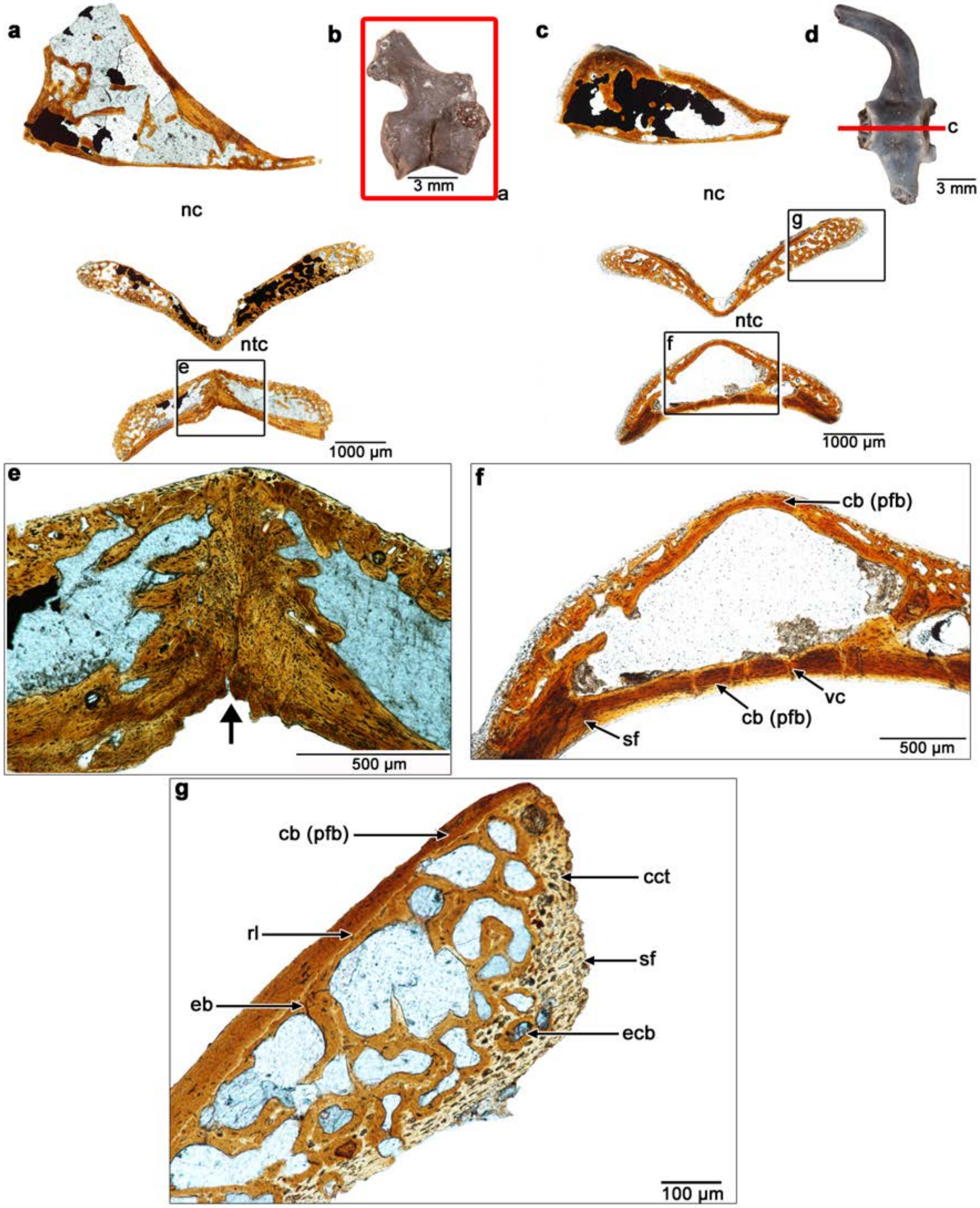

Extended Data Figure 1. Additional vertebrae sectioned for this study. **a**, sagittal section of a caudal vertebra (ROM 73772) with visible fracture plane (**b**). **c**, sagittal section of an anterior, rib-bearing caudal vertebra (ROM 77410) lacking a fracture plane (**d**). **e**, closeup of ventral centrum wall in the sagittal section of ROM 73772 showing position of fracture plane. **f**, closeup of ventral centrum wall in the sagittal section of ROM 77410 showing the lack of a fracture plane within the cortical bone. **g**, closeup image of major tissue types of the centrum (taken from ROM 73774). Arrow indicates position of

fracture plane in thin section. Abbreviations: cb (pfb), cortical bone (parallel-fibered bone); cct, calcified cartilage; eb, endosteal bone; ecb, endochondral bone; nc, neural canal; ntc, notochordal canal; rl, reversal line; sf, Sharpey's fibers; vc, vascular canal.

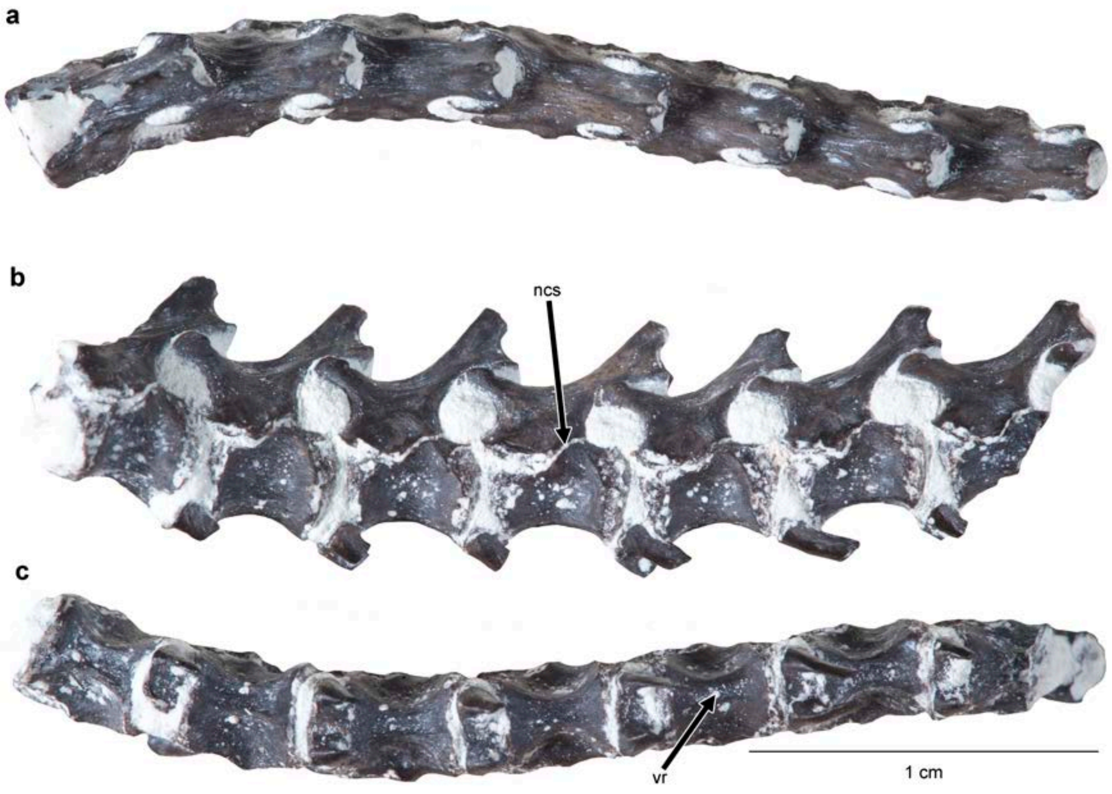

Extended Data Figure 2. Series of seven haemal arch bearing caudal vertebrae belonging to an unidentified parareptile (identification based on presence of ventral keels on caudal centra) from the Early Permian Richards Spur locality (ROM 77622). Note the absence of fracture planes on the centra. Open neural central sutures indicate that this is a very young individual. **a**, dorsal view; **b**, lateral view; **c**, ventral view. Abbreviations: ncs, neural central suture; vr, ventral ridge.
